# On the performance of de novo pathway enrichment

**DOI:** 10.1038/s41540-017-0007-2

**Published:** 2017-03-03

**Authors:** Richa Batra, Nicolas Alcaraz, Kevin Gitzhofer, Josch Pauling, Henrik J. Ditzel, Marc Hellmuth, Jan Baumbach, Markus List

**Affiliations:** 1 Department of Mathematics and Computer Science, University of Southern Denmark, Odense, Denmark; 2 Institute of Computational Biology, Helmholtz Zentrum München, Munich, Germany; 3 Department of Dermatology and Allergy, Technical University of Munich, Munich, Germany; 4 Department of Cancer and Inflammation Research, Institute of Molecular Medicine, University of Southern Denmark, Odense, Denmark; 5 Center for Bioinformatics, Saarland University Campus, Saarbrücken, Germany; 6 Department of Biochemistry and Molecular Biology, University of Southern Denmark, Odense, Denmark; 7 Department of Oncology, Odense University Hospital, Odense, Denmark; 8 University of Greifswald, Institute for Mathematics and Computer Science, Greifswald, Germany; 9 Computational Systems Biology group, Max Planck Institute for Informatics, Saarland Informatics Campus, Saarbrücken, Germany; 10 Computational Biology and Applied Algorithmics, Max Planck Institute for Informatics, Saarland Informatics Campus, Saarbrücken, Germany

## Abstract

De novo pathway enrichment is a powerful approach to discover previously uncharacterized molecular mechanisms in addition to already known pathways. To achieve this, condition-specific functional modules are extracted from large interaction networks. Here, we give an overview of the state of the art and present the first framework for assessing the performance of existing methods. We identified 19 tools and selected seven representative candidates for a comparative analysis with more than 12,000 runs, spanning different biological networks, molecular profiles, and parameters. Our results show that none of the methods consistently outperforms the others. To mitigate this issue for biomedical researchers, we provide guidelines to choose the appropriate tool for a given dataset. Moreover, our framework is the first attempt for a quantitative evaluation of de novo methods, which will allow the bioinformatics community to objectively compare future tools against the state of the art.

## Computational biology: Evaluation of network-based pathway enrichment tools

*De novo* pathway enrichment methods are essential to understand disease complexity. They can uncover disease-specific functional modules by integrating molecular interaction networks with expression profiles. However, how should researchers choose one method out of several? In this article, a group of scientists from Denmark and Germany presents the first attempt to quantitatively evaluate existing methods. This framework will help the biomedical community to find the appropriate tool(s) for their data. They created synthetic gold standards and simulated expression profiles to perform a systematic assessment of various tools. They observed that the choice of interaction network, parameter settings, preprocessing of expression data and statistical properties of the expression profiles influence the results to a large extent. The results reveal strengths and limitations of the individual methods and suggest using two or more tools to obtain comprehensive disease-modules.

## Introduction

Modern high-throughput OMICS technologies (genomics, transcriptomics, proteomics, metabolomics, etc.) are driving the exponential growth of biological data. With continuously decreasing costs, it is now possible to determine the activity of genes, proteins and metabolites, their chemical modification (phosphorylation, methylation, etc.), as well as mutations on a genome scale. For example, the gene expression omnibus (GEO) alone hosts >51,000 study records derived from >1600 organisms.^1^ Moreover, many international consortia have gathered data from thousands of samples, for instance, the International Cancer Genome Consortium^2^ has collected data on 50 cancer types derived from several OMICS technologies. To gain a holistic view of such huge datasets, network-based analysis has become a promising alternative to traditional enrichment approaches such as gene ontology enrichment^3^ or gene set enrichment analysis.^4^ The objective is to study condition-specific systemic changes by projecting experimental data on network modules, representing conditionally perturbed biological processes. Despite the inherent noise and incompleteness of currently available networks, network-based analysis has compelling advantages. It can capture the modular interplay of biological entities and processes overlooked in traditional enrichment methods^5^ and can further unravel molecular interactions not covered in well-defined pathways.^6^ In addition, network- based pathway enrichment methods can unravel crosstalk between pathways or sub-mechanisms which remain undetected in traditional pathway enrichment methods with known pathways such as KEGG or Reactome.^7^

### De novo pathway enrichment

Over the years, several approaches have been proposed for network-based analysis of experimental data. While some methods exploit network topology to augment scoring of known pathways^4, 8–16^, others rely on experimental data to reconstruct a condition-specific interaction network^17–20^. Here, we focus on methods that perform de novo pathway enrichment. These methods integrate experimental data with a large-scale interaction network, to extract sub-networks enriched in biological entities active in a given experimental dataset. The definition of active depends on the type of dataset, for instance, differential expression in the case of gene expression data.

**Fig. 1:**
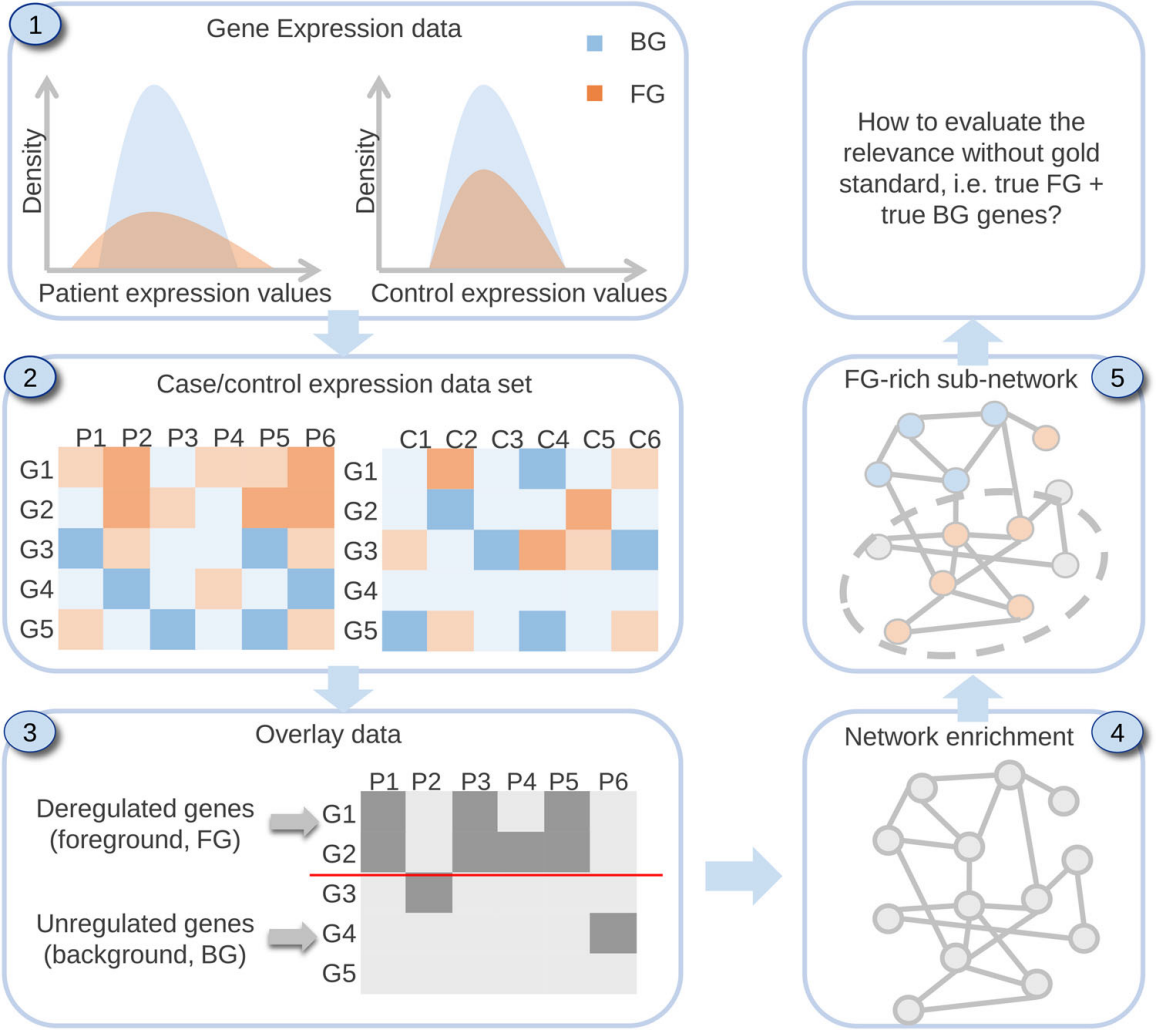
A typical workflow for de novo pathway enrichment. The underlying hypothesis is that phenotype-specific genes (foreground, FG) are differentially expressed in many case samples compared to a control group (**1**, **2**) By using statistical tests, one can determine which genes are affected by the phenotype (**3**) and overlay this information on an interaction network (**4**) De novo pathway enrichment tools aim to extract sub-networks enriched with phenotype-specific FG genes (**5**) Comparing several such methods is an open issue

Diverse strategies for de novo pathway enrichment methods have been devised. To summarize the state of the art, we classify existing methods into four categories (Supplementary Fig. 1): (1) aggregate score optimization methods, (2) module cover approaches, (3) score propagation approaches, and (4) clustering-based approaches. Detailed descriptions of these classes are provided in the supplementary material. While several such methods exist (see Table 1), there are no guidelines to aid potential users in the choice of the appropriate tool. To fill this gap, we present a comparative analysis of selected tools across different datasets and parameter settings, as well as propose the first gold standard for quantitative benchmarking of de novo pathway enrichment methods (Fig. 1).

**Table 1:** List of publicly available de novo network enrichment methods in alphabetical order (February 7, 2016)

Tool	Method	Software	Reference
BioNet*	ASO	app	Ref. 25
ClustEx	Clust.	app	Ref. 32
cMonkey	Clust.	app	Ref. 33
COSINE*	SP	app	Ref. 26
GiGA*	SP	app	Ref. 34
GXNA*	SP	app	Ref. 29
HotNet	SP	app	Ref. 35
jActiveModules	ASO	C-PL	Ref. 36
KeyPathwayMiner*	MC	app, C-PL, WS	Ref. 30
DEGAS*	MC	app	Ref. 37
MEMCover	MC	app	Ref. 38
NetWalker	ASO	app	Ref. 16
NetworkTrail	ASO	WS	Ref. 39
PinnacleZ*	ASO	app, C-PL	Ref. 31
ReactomeViz-MCL	Clust.	C-PL	Ref. 40
RegMOD	SP	app	Ref. 41
ResponseNet	ASO	WS	Ref. 42
SubExtract	ASO	app	Ref. 43
TieDIE	SP	script (Python)	Ref. 44

De novo methods capable of processing gene expression data are sub-divided into four categories, i.e. *ASO* aggregate score optimization, *SP* score propagation, *MC* module cover and *Clust* clustering based; see supplementary material for details. The availability of the tool as a stand-alone application (app), as Cytoscape plugin (C-PL), or as web service (WS) is further indicated.

* Included in our quantitative study (see text for details)

### Challenges in evaluating de novo pathway enrichment tools

Pathway enrichment methods belong to the family of unsupervised statistical learning algorithms. In this context, unsupervised means that the quality of potential solutions cannot be assessed objectively unless the optimal solution is known a priori, i.e., a gold standard is needed.

In case of de novo pathway enrichment, such a gold standard consists of genes known to be relevant (or irrelevant) to the condition(s) measured in the experimental data. In other words, a set of foreground genes (FG) and a set of background genes (BG) are required, such that one can utilize the proportion of FG genes (relevant) compared to BG genes (irrelevant) in the extracted sub-networks as a performance measure. In the following, we discuss why such a gold standard is currently unavailable.

The disease- specific pathways of the KEGG database^21^ could be considered as a potential gold standard, as they cover many known genes (and their interplay) associated with a certain disease or biological function. However, these genes constitute an incomplete FG gene set, since it is unlikely that all disease-associated genes are known. Furthermore, there is no distinction of potential FG genes and BG gene sets. This bias prohibits a fair and robust evaluation of de novo pathway enrichment tools using such databases.

To address this issue, we propose a protocol to generate artificial, bias-free gold standard FG and BG datasets. Our approach relies on two main assumptions:

#### Signal strength

Signal strength also known as signal to noise ratio, it describes the dissimilarity of the expression values for a given set of FG genes compared to the remaining BG genes. The higher the signal strength, the more diverse are the expression profiles of the FG genes compared to the BG genes. Thus, we can expect pathway enrichment methods to identify them more easily.

#### Sparsity

It models the average distance between FG genes in the graph. The lower the sparsity, the more densely the FG genes are distributed over the network, such that pathway enrichment methods are expected to find them more easily.

#### Analysis strategy

We systematically generated test expression datasets with preselected (synthetically) FG and BG genes sets. Subsequently, representative de novo network enrichment tools were applied to identify FG-rich (and BG-poor) modules across parameter sets for each tool and for varying problem complexities, i.e. different levels of signal strength and sparsity. We used two common protein-protein interaction networks, namely the human protein reference database (HPRD)^22^ and the interlogous interaction database (I2D).^23^

## Results

### The effect of varying signal strength

**Fig. 2:**
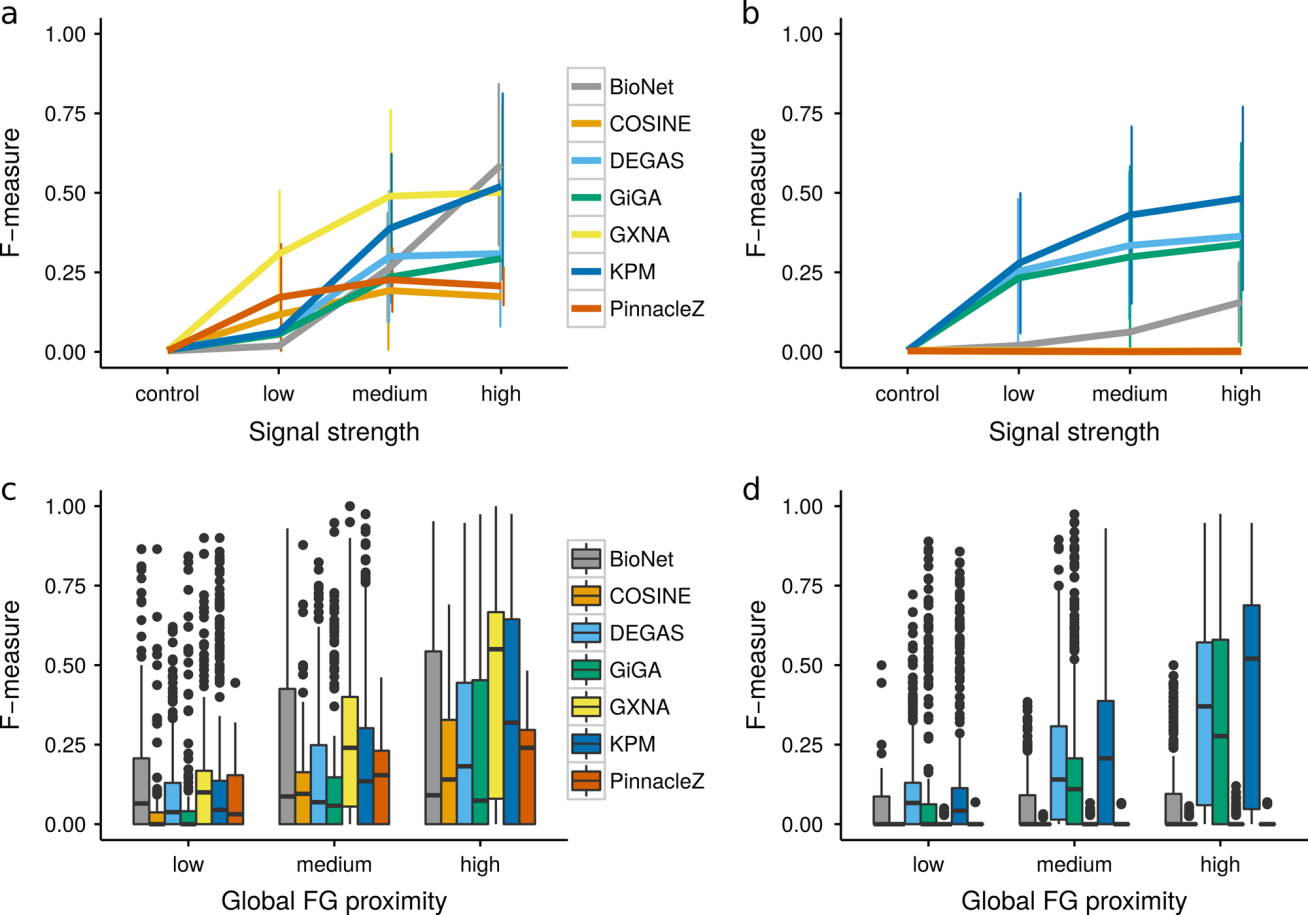
Average performance for over 80 FG sets of size 20 nodes were generated, using the AVD_*k*_ algorithm, with varying signal strength (**a**, **b**) and varying sparsity (**c**, **d**). Expression profiles were simulated with varying mean (VM) (**a**, **c**) and varying variation (VV) (**b**, **d**). The HPRD network was used as the input network and the performance was assessed using the F-measure. The *error-bars* (**a**, **b**) and *box plots* (**c**, **d**) represent performance over several FG nodes and over a range of internal parameter settings for each tool. The higher the signal strength, the more different are the expression profiles of the FG vs. BG genes, indicating that we can expect pathway enrichment methods to identify them more easily. For details on internal parameters, signal strength and sparsity values, please refer to Supplementary Tables 1, 3, 5 respectively

We generated expression profiles with varying mean (VM) and varying variation (VV) (see Fig. 4 and “Methods” for details). Figure. 2a and b illustrate that with increasing signal strength generally improves the performance of all tools.

BioNet, GXNA, and KeyPathwayMiner (KPM) performed best with VM. However, if the simulation of expression profiles was based on changes in the variance (VV) between FG and BG distributions, the top performers BioNet and GXNA performed poorly. We speculate that this was due to their internal expression profile preprocessing procedures. Thus, one should carefully consider the characteristics of FG and BG expression profiles in the choice of the most suitable tool.

### The effect of varying sparsity

We used two algorithms to distribute the label FG across the nodes in the network. The seed-and-extend (SAE) algorithm generates connected random sub-networks of a particular size and labels all its nodes as FG. In contrast, the average-distance-k (AVD_*K*_) algorithm distributes the FG node labels such that their average pairwise distance equals a given *k*. With increasing *k* values, the sparsity grows. All other nodes in the network were labeled as BG. We computed the sparsity of the FG nodes with three measures (a) Global FG proximity: Shortest path covering the FG set nodes in the network. (b) Global FG connectivity: Average degree of the FG nodes in the network. (c) Local FG density: Graph density of the FG nodes.

As expected and depicted in Fig. 2c and d, the performance of all tools decreased with increasing sparsity (decreasing FG proximity, i.e. increasing *k* using AVD_*k*_). Supplementary Fig. 2 further illustrates the performance dynamics with increasing FG density and connectivity. Evidently, all tools performed better with FG nodes selected using the SAE method (Supplementary Fig. 3). However, biological processes are complex and interaction networks are incomplete. Genes that are deregulated in a condition/disease may not be directly connected in the cellular network. Consequently, we believe that generally AVD_*k*_ is a better, more natural choice for FG selection. The results for the I2D network are depicted in Supplementary Figs 4 and 5.

### The effect of varying internal parameters

**Fig. 3:**
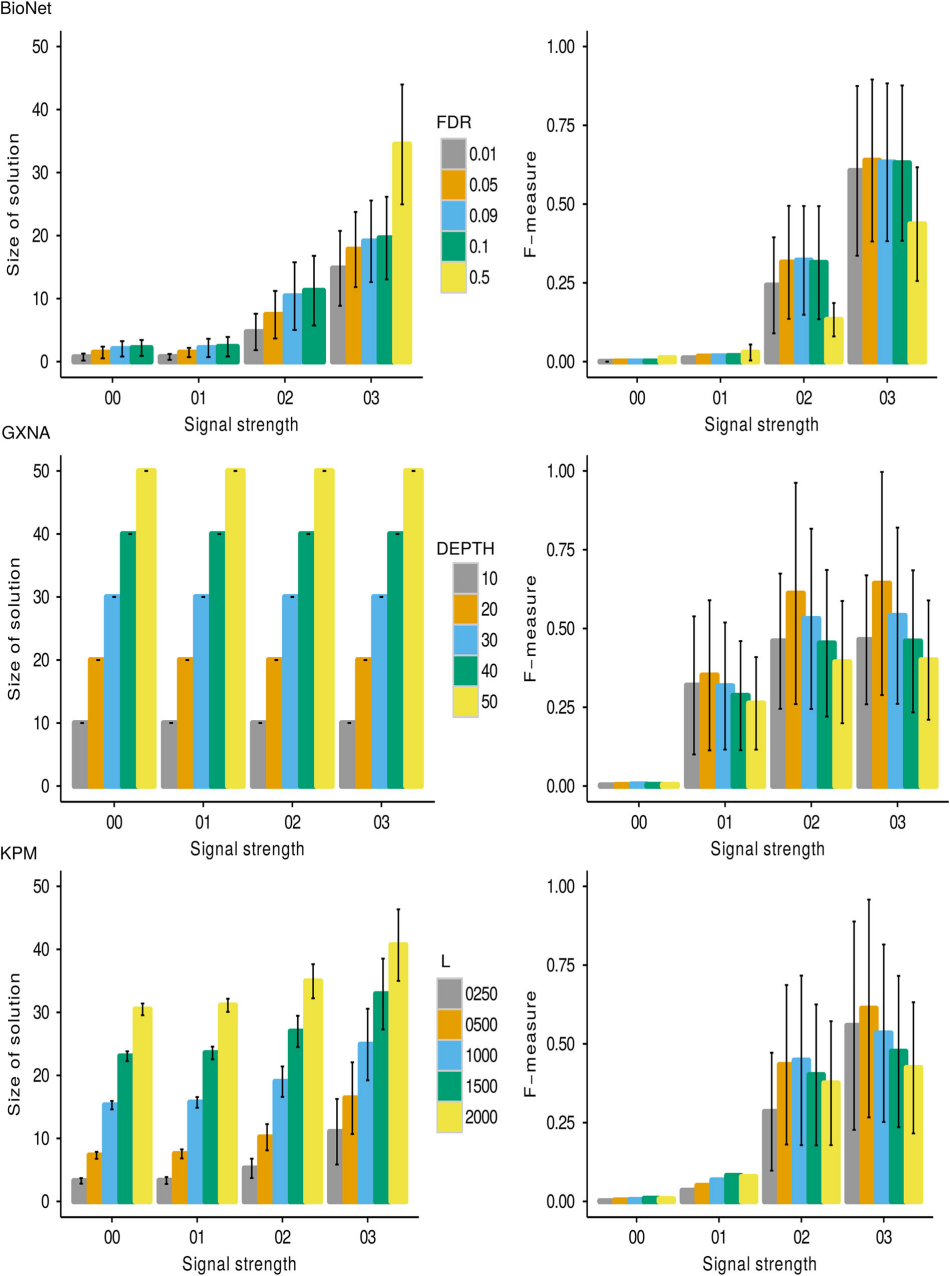
Average performance for over 80 FG sets of size 20 nodes generated using the AVD_*k*_ algorithm. Expression profiles were simulated with VM. The HPRD network was used as the input network. Performance was assessed with the F-measure for a range of internal parameter settings for each tool, i.e. the expected pathway size (M) for GXNA, the number of allowed exceptions (outliers) in a pathways (L) for KPM, and the pathway false discovery rate (FDR) for BioNet

We can further categorize tools based on the characteristic of their internal parameters (IP). COSINE, GiGA, GXNA, and PinnacleZ restrict the solution to a user-specified size. In contrast, BioNet, DEGAS and KPM allow for noise (exceptions or outliers) in the reported solutions, such that sub-networks can have arbitrary size within certain constraints to allow for noise. We tested each selected method on a wide range of IP values. Overall, the trend was that with increasing IP values the size of the solution increases (Fig. 3). Lower IP values led to solutions with fewer nodes, whereas higher IP values led to larger sub-networks. As expected, the performance (F-measure) decreased towards the extreme ends of the IP range.

### Summary

Consistently across the two tested networks (HPRD and I2D), BioNet, COSINE, GXNA, and PinnacleZ performed better with expression data generated using VM as compared to VV. DEGAS yielded better results with VV, while the performance of KPM and GiGA was independent of VV and VM. When using VM GiGA, BioNet, GXNA, and KPM showed better performance with SAE (dense FG nodes) compared to AVD_*K*_ (relatively sparse). COSINE, DEGAS, and PinnacleZ appeared less affected. These trends were conserved across the two networks. Using HPRD as a network base, KPM, DEGAS, and GiGA performed better with VV with both FG node selection algorithms. All tools performed poorly with VV and AVD_*K*_ when using I2D. However, when using SAE instead, KPM and GiGA performed very well.

Based on our analysis, we propose the following guidelines for biomedical researchers, (a) if computationally feasible, choose a comprehensive biological network such as I2D for capturing a wide range of known or predicted biological interactions (b) apply several tools to obtain a comprehensive picture of the information captured in the dataset. In any case, a wide range of IP should be tested. Please note that apart from gene expression profiles, some tools can be used for other types of OMICS data (molecular profiles) such as protein expression profiles or RNA sequencing data.

## Discussion and conclusion

De novo pathway enrichment is a powerful method to uncover condition-specific functional modules in molecular biological networks. Existing methods for integrating networks and experimental data have recently been reviewed in Mitra et al.^6^ Nevertheless, systematic and quantitative evaluations of the performance of existing methods and methodology to do so, have yet to be published.

To establish a basis for such a comparison in the absence of a suitable gold standard, we propose a strategy to assess the performance of de novo pathway enrichment tools on simulated data. We sampled nodes in the network (e.g. genes) that are relevant in a condition from a foreground distribution and non-relevant nodes from a background distribution. By adjusting the sparsity and signal strength of the solutions “hidden” in the network, we were able to adjust the difficulty of the de novo pathway enrichment problem and systematically compare the performance of selected tools.

We compared seven tools, representing diverse methodological approaches, to study the influence of data preprocessing and parameter choice on the quality of the solutions. In our analysis, BioNet and KPM often performed best, presumably because these tools leave the preprocessing (computation of *p*-values from expression data, for instance) to the user. Our results show that, in absence of a well-defined gold standard, using two or three different tools can provide a comprehensive picture of the information content of the data. In the future, we hope to extend our framework to a platform for meta or ensemble analysis using several tools in conjunction. Such a method could, for instance, combine results from different tools into concatenated (sub-)networks.

The framework presented here allows for the first standardized, well-structured and comprehensive evaluation of de novo pathway enrichment methods. However, the design of an optimal benchmark scheme relies on reasonable model assumptions and is a challenging task. Our conclusions are limited by nature, as we have no a priori gold standard datasets. While our knowledge of disease relevant genes continuously improves, we still lack sets of non-relevant genes in certain diseases. Nevertheless, we may assume that disease genes are generally closer to each other in a molecular biological network,^24^ and that their expression (in general) is differentially distributed compared to non-disease genes. Considering this, we believe to offer a reasonable test scenario for de novo pathway enrichment methods. However, we acknowledge that existing methods are diverse in methodology as well as in their expected input datatypes and formats. As their performance and ranking varies greatly with their IP and the characteristics of the expression datasets, all rankings should be carefully interpreted. While simulated data are suitable for objective comparisons, we acknowledge that we cannot fully model the complexity of biological systems. A promising way forward would be to use large-scale perturbation experiments, such as shRNA or CRISPR knockdown/knockout studies, in a benchmark.

## Methods

### Candidate tool selection

For our analysis, we selected methods available as standalone applications, a prerequisite for a systematic and automated analysis across different datasets and parameters. We further restricted our comparison to tools designed for pathway enrichment using gene expression data, since it is the most common application type. The following seven tools fulfill both inclusion criteria: (a) BioNet^25^ is an aggregate score optimization method that implements an efficient integer linear programming approach to compute optimal sub-networks. (b) COSINE^26^ is a score propagation method that includes not only nodes but also edges in its scoring function. Edges correspond to differential gene–gene co-expression across different groups. (c) DEGAS^27^ is a module cover approach that uses a shortest path heuristic to identify sub-networks with at least *K* “active” nodes, with “active” being defined via the proportion of active cases in the experimental data. (d) GiGA^28^ is another score propagation method that first computes local minima to serve as starting points for iteratively building sub-networks with *n* members and a maximal rank of *m*. (e) GXNA^29^ is a score propagation method that selects random nodes as seeds of candidate sub-networks. These are iteratively extended by adding the neighboring node with the highest score. (f) KPM^30^ is another module cover approach inspired by DEGAS. It heuristically extracts maximal connected sub-networks, where all nodes except for at most *K* are differentially expressed in a given set of samples but at most *L*. (g) PinnacleZ^31^ is an aggregate score propagation method that extends a set of random seed nodes to a number of sub-networks, which are then filtered by comparing their score distribution with that of randomly generated sub-networks. We provide more details for each of these tools in the supplementary material and briefly summarize some application cases. Please note that due to unavailability of a standalone application, clustering-based approaches could not be considered in our analysis.

### The network data

The human protein–protein interaction (PPI) networks from the HPRD^22^ and the I2D^23^ were selected as test networks. The HPRD network consists of 9425 nodes and 36,811 edges. I2D network consists of 17,351 nodes and 217,379 edges. The networks could be modeled as undirected graphs *G* consisting of a set of vertices *V* and a set of edges *E*. To gain insights into how network complexity affects the performance of the selected tools, we chose two networks that differ largely in network complexity, e.g. recent interaction networks such as I2D are orders of magnitude larger than classical PPI networks such as HPRD.

### Simulation of gene expression datasets

For each graph *G*, we split the nodes into FG and BG genes. Next, we simulated expression profiles for a set of 110 samples, 100 patient samples and 10 controls. Please note, we have chosen an arbitrary number of samples in each class. This choice can also be guided by (a) random sampling or (b) GEO GDS datasets, in order to reflect the median-experiment size in real case studies. We lack it in our current study due to limited time and computational power. We will work on this in the future, even though we expect the main findings of our study to remain unaffected.

We divided the simulation into three cases: (i) of patient-FG, (ii) of control-FG, (iii) of BG for patients and controls. We used the following models for the simulation:

#### (a) Varying variance

**Fig. 4:**
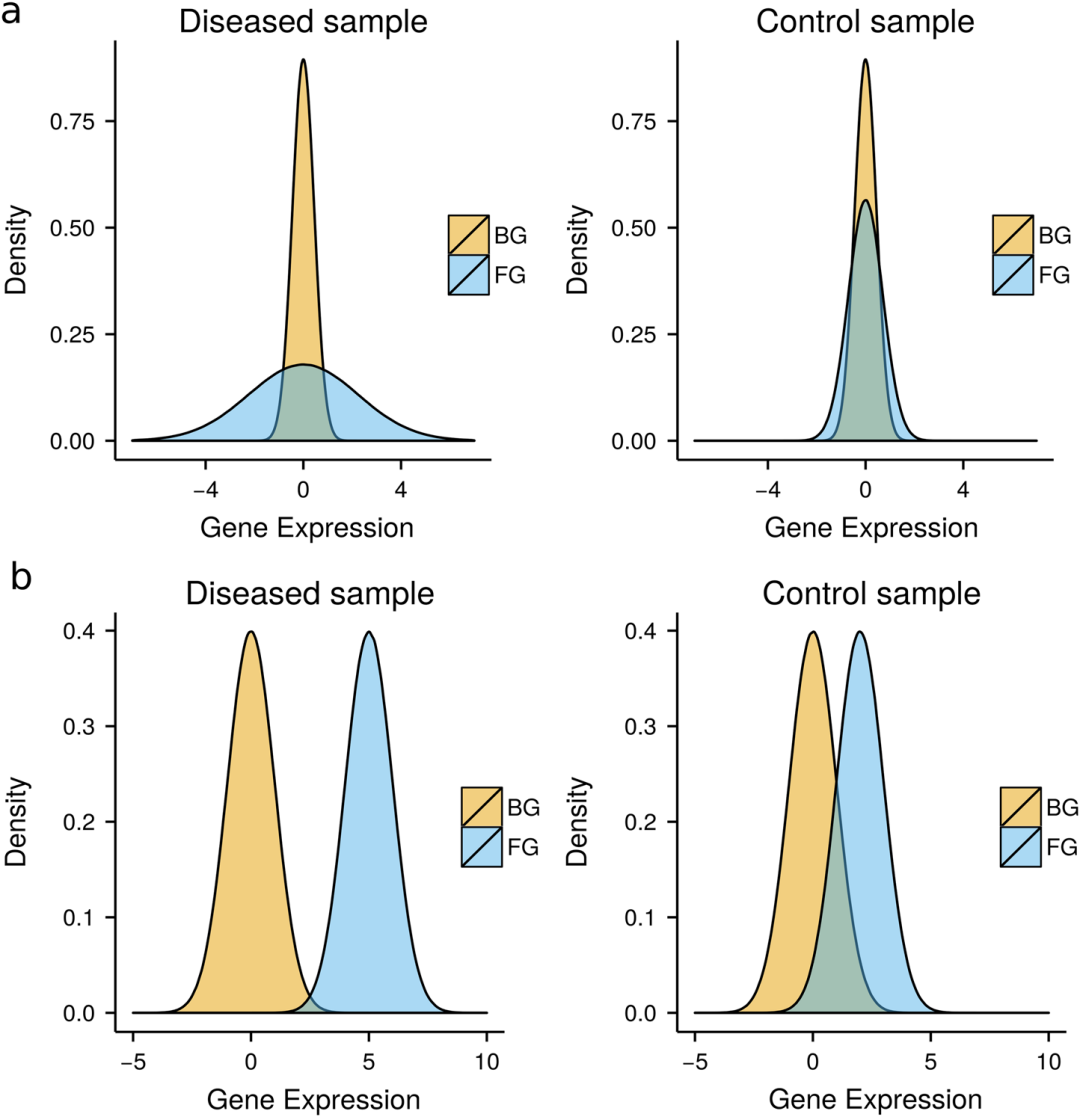
Illustration of the used models for FG and BG expression distributions generated for cases and control samples: VV in (**a**) and VM in (**b**)

We simulated the expression profiles for the patient-FG genes and—BG genes from normal distributions *N*(*μ, v*_BG_) ∧ *N*(*μ, v*_BG_) respectively (Fig. 4a). We used the same distribution for (ii) and (iii). *μ* *=* *0* was used in all cases. Signal strength was defined by the ratio *v*_FG_, *v*_BG_. See Supplementary Table 3 for the specific values selected for variances *v*_FG_, *v*_BG_ in each scenario.

#### (b) Varying mean

In this case, we simulated the expression profile for the FG genes from a normal distribution *N*(*μ*_FG_, *v*), while for the expression values for BG genes we used *N*(*μ* *=* 0, *v*) and *N*(*μ*_FGC_, *v*) for control-FG (Fig. 4b). Here, we used the setting *v* *=* 1 in all cases. Signal strength was defined by the difference *μ*_FG_−*μ*_FGC_. See Supplementary Table 2 for the specific values selected for means *μ*_FG_, *μ*_FGC_.

We further transformed the raw data into tool specific input, (e.g. *p*-values), the particular pre-processing steps for each tool are specified in the supplementary material.

### Selection of FG and BG nodes

We used two approaches for splitting a given graph *G* *=* (*V*, *E*) into FG and BG nodes.

#### Seed and extend approach

This is a simple approach, in which we (a) randomly selected a seed node *s*, (b) expanded the sub-graph by including the nodes from the neighborhood of *s*, and (c) continued expanding the neighborhood until the sub-graph *s* had *n* *=* 20 nodes or until the number of nodes in the sub-graph remains the same as *i*−1 iteration. If the sub-graph exceeds *n,* we chose *n−*1 neighbors randomly from the set of neighbors.

#### AVD_*k*_

In this approach, the sparsity is modeled for a set of FG nodes in a graph as the average pairwise shortest distance between them. By generating FG node sets of varying distance *k,* the sparseness of the sub-networks (or closeness of the FG genes) could be adjusted. Given a network modeled as graph *G* and a sparsity level *k*, we define the problem of selecting the FG nodes, as the average distance *k *± *α* problem, or AVD_*K*_ for short. The goal was to identify a set of nodes from *W* ⊆ *V* such that the average shortest distance between all nodes of *W* have an average shortest distance (in *G*) to each other of *k *± *α*. If not stated otherwise, we set *α* *=* 1. Our sparseness function ADV_3_, for instance, would thus return a set of nodes (which we will later on treat as FG nodes) having, on average, a pairwise shortest distance to each other between 2 and 4. We introduced *α* parameter to allow for inexactness and variability. Finding solutions for AVD_*K*_ is computationally difficult (NP-complete). We first tackled it by using integer-linear programming, but it proved too slow and memory intense for graphs with thousands of nodes and edges (see Supplementary Material). To deal with the size of current biological networks, we developed a greedy heuristic running in polynomial time to compute solutions for the AVD_*K*_ problem. The pseudo-code and additional details can be found in the supplementary material.

#### Availability

All **s**cripts and data used in this study are available online at http://patheval.compbio.sdu.dk.

## Electronic supplementary material

Supplementary Information
