# Supplementary material for: On the performance of de novo pathway enrichment
Source: NPJ Syst Biol Appl. 2017 Mar 3;3:6. doi: 10.1038/s41540-017-0007-2 (PMC5445589; doi:10.1038/s41540-017-0007-2)
Supplement: Supplementary file 1 — Supplementary Information [file 41540_2017_7_MOESM1_ESM.pdf]

On the performance of de novo pathway enrichment Supplementary Material

Richa Batra*[1,9,10], Nicolas Alcaraz[1,3], Kevin Gitzhofer[2], Josch Pauling[7], Henrik Ditzel[3, 8], Marc Hellmuth[2,4], Jan Baumbach †‡[,1,5], and Markus List‡[,6]

[1]Department of Mathematics and Computer Science, University of Southern Denmark, Odense, Denmark

[2]Center for Bioinformatics, Saarland University, Saarbrücken, Germany

[3]Department of Cancer and Inflammation Research, Institute of Molecular Medicine, University of Southern Denmark, Odense, Denmark

[4]University of Greifswald, Institute for Mathematics and Computer Science, Greifswald, Germany

[5]Computational Systems Biology group, Max Planck Institute for Informatics, Saarbrücken, Germany

[6]Computational Biology and Applied Algorithmics, Max Planck Institute for Informatics, Saarbrücken, Germany

[7]Department of Biochemistry and Molecular Biology, University of Southern Denmark, Odense, Denmark

[8]Department of Oncology, Odense University Hospital, Odense 5000, Denmark

[9]Institute of Computational Biology, Helmholtz Zentrum München, Munich, Germany

[10]Department of Dermatology and Allergy, Technical university of Munich, Munich, Germany

‡joint last author

*richa.batra@helmholtz-muenchen.de

†jan.baumbach@sdu.dk

Contents

1 Classical enrichment methods 3

2 *De novo* pathway enrichment methods and their classification 4

3 Selected *de novo* pathway enrichment tools 5

4 Methods for generating evaluation datasets 8

5 Supplementary Tables and Figures 11

1 Classical enrichment methods

In biomedical settings, after appropriate statistical tests are performed, lists of differentially expressed (or mutated or phosphorylated) genes emerge from molecular profiles of the samples. Ideally, all these candidate genes, can be validated in the wet lab. However, it has become a standard to account previous biological knowledge before further experiments are conducted. It is to obtain more confidence on the results, gain further insight or to simply reduce the candidate list.

The biological knowledge commonly used are: a) gene functional categories, typically from Gene Ontologies [1], b) gene sets: consisting of collected genes related to the same biological process, can be custom built gene sets or from curated databases such as the Molecular Signatures Database (MSIG)[2], or c) pathways: consisting of sets of genes and their interactions (gene regulations, protein-protein interactions, metabolic reactions, etc), usually obtained from expert-curated pathway databases such as KEGG [3], REACTOME [4] and BioCyc [5], to name some examples.

Furthermore, these integrative methods are usually coined as "enrichment" procedures, and can be classified into *over-representation* and *aggregate score* methods. Over-representation methods start out from the preliminary candidate list obtained from traditional differential expression analyses. Afterwards, for each functional category, gene set or pathways, their overlaps with the candidate genes are stored in contingency tables. Finally, statistical tests such as the χ^2-test, hypergeometric or binomial distributions are applied to assess the over-representation. It's important to note that over-representation analysis do not incorporate genes that did not make it to the final candidate gene list, hence are highly dependent on the methods and cutoff applied to obtain the candidate gene list. Example tools or web-services that perform over-representation analysis are GOStats [6], DAVID [7], PANTHER [8] and ConsensusPathDB [9].

On the other hand, aggregate score methods, start out from the entire gene list and the expression values, either raw or transformed (fold changes, p-values, etc). Here, the aim is to produce a summary score for each functional category, gene set or pathway. In the case of gene sets, the most popular method is Gene Set Enrichment Analysis (GSEA) [2], which is based on the Kolmogorov–Smirnov (K–S) test. Several other methods have emerged, such as Significance Analysis of Functional categories in gene Expression studies (SAFE) [10], Generally Applicable Gene set Enrichment (GAGE) [11], Mean-Rank Gene Set Enrichment tests (MRGSE) [12], among others. Some variants produce a score for each sample, single-sample GSEA (ssGSEA)[13], and Gene Set Variation Analysis (GSVA [14]). For a comparative study of the performance of such methods, we refer to the work by Tarca et. al. [15].

In the case that aggregate methods are applied to pathways, usually named "Pathway Enrichment", techniques have emerged that take the topological information into account in the scoring function. TAPPA [16], for example, defines a pathway-activity score, based on all connected gene pairs in the pathway. While Hung et al. compute gene weights based on their correlated neigh-

bors [17]. Other methods score pathway activity with random walks [18,19], where Liu et al. additionally consider directed edges [20]. A more focused method is SPIA [21], which takes inspiration from Google's PageRank algorithm [22] to determine the influence of a gene in a signaling pathway and to compute an impact score for the whole pathway. In case evidence from different sources/datatypes (e.g. expression, mutations, etc.) are available, PARADIGM [23] is able to integrate several OMICS by means of probabilistic graphical models to infer the degree to which a pathway's activities are altered in, for instance, cancer patients.

2 *De novo* pathway enrichment methods and their classification

In contract to classical pathway enrichment methods, *de novo* pathway enrichment methods have been proposed to search for novel pathways that are not contained in a predefined list. Note that by *de novo* we exclude network-based analyses such as network inference (refer to [24] for a review of these), where the objective is to reconstruct a complete network structure from the molecular profiles. Instead, we specifically refer to the integration of biological networks (such as protein-protein interactions (PPI), gene regulatory (GRN), or metabolic networks) with functional activity profiles (such as gene expression, mutation, protein phosphorylation, etc.) to extract sub-networks that are enriched with differentially regulated biological entities. We group *de novo* pathway enrichment methods into the following distinct categories:

(i) **Aggregate score optimization approaches:** These methods, pioneered by Ideker et al. [25], search for connected sub-networks with maximal score. The score summarizes the level of activity of genes in the pathway. This method starts out by defining a score for individual genes, such as the raw profile values or adjusted p-values from, for example, case-control studies. Some methods extend this notion to edge-scores, such as the pairwise correlation of genes [26] or co-expression p-values [27]. Afterwards, an appropriate aggregate scoring function for the set of genes is defined. The aggregate function can be based on combining multiple p-values [25], mutual information [28], or signal content [29]. After mapping each gene to a node in the network, the objective is to extract connected sub-networks that maximize (minimize) the aggregate score. All of these methods have in common that the underlying optimization problem translates to a NP-Hard problem. Most tackle the computational hardness with heuristics such as greedy methods [28,30], simulated annealing [25] or genetic algorithms [31]. However, exact methods that are able to provide optimal solutions in reasonable time have also been implemented [29,32]. Since the search method relies heavily on the scoring function (which assumes a particular distribution of the data), these *de novo* pathway enrichment methods are typically restricted to a certain type of OMICS measurement such as gene expression data or mutation profiles. However, some methods [31] have extended these approaches to other types of datasets as well.

(ii) **Score propagation approaches:** A different type of *de novo* pathway enrichment method

extracts sub-networks by first propagating individual gene-scores through the network. Strategies to propagate the values include diffusion-flow methods [33], where gene scores ”flow” through the networks similar to heat through pipes. Other strategies are based on random walks [34], where gene scores are iteratively recomputed based on the scores of their neighbors, similar to how the PageRank [22,35] algorithm computes the importance of web-pages for search engines. Other approaches propagate scores in a more indirect way, by incorporating network information into regression models [36] and adjusting the gene-scores based on their ability to distinguish between two or more phenotype labels (e.g. case-control, cancer-subtypes). Independently of the propagation strategy, all methods eventually report a set of connected components consisting of all or a certain number of genes above a certain score cutoff.

(iii) **Module cover approaches:** These approaches [37–39] consider the statistics to determine relevant genes/samples as a separate pre-processing step. This allows for an appropriate method to be selected to determine differential activity for each individual gene or sample based on the properties of the dataset. Afterwards, the objective of these methods is to extract connected sub-networks that ”cover” a certain (maximum, or minimum) number of significantly active genes and/or samples. Similar to approaches of type (i), these models are also computationally intensive, since they relate to NP-Hard optimization problems such as the set-cover problem.

(iv) **Clustering based approaches**: This group of methods applies known clustering techniques into their workflow. The goal is to extract connected sub-networks that contain genes showing similar patterns in their molecular profiles. Methods can either apply traditional network clustering, where the edge weight reflects the similarity of the genes in the molecular profile [40], or apply network clustering strategies to directly cluster differentially active genes in the network while ensuring module connectivity on an extra step [41]. These *de novo* pathway enrichment cluster methods can be distinguish from other integrative clustering approaches such as biclustering [42] (co-clustering or two-way clustering) efforts, which cluster genes based on their molecular profiles, but also integrate network connectivity into the similarity function. While the objective of these methods [43,44] is also to cluster genes into groups that may represent functional modules, these are not necessarily connected sub-networks. Similar to traditional clustering methods, cluster based *de novo* pathway enrichment requires an input parameter that determines the number of clusters.

3 Selected *de novo* pathway enrichment tools

For our evaluation analysis, we selected tools satisfying the following conditions: a) implemented in free non-commercial software. b) standalone or library version available for easy batch scripting, c) able to accept an arbitrary interaction network, and d) designed to deal with gene expression data. Thus, our final list for evaluation consists of 7 tools:

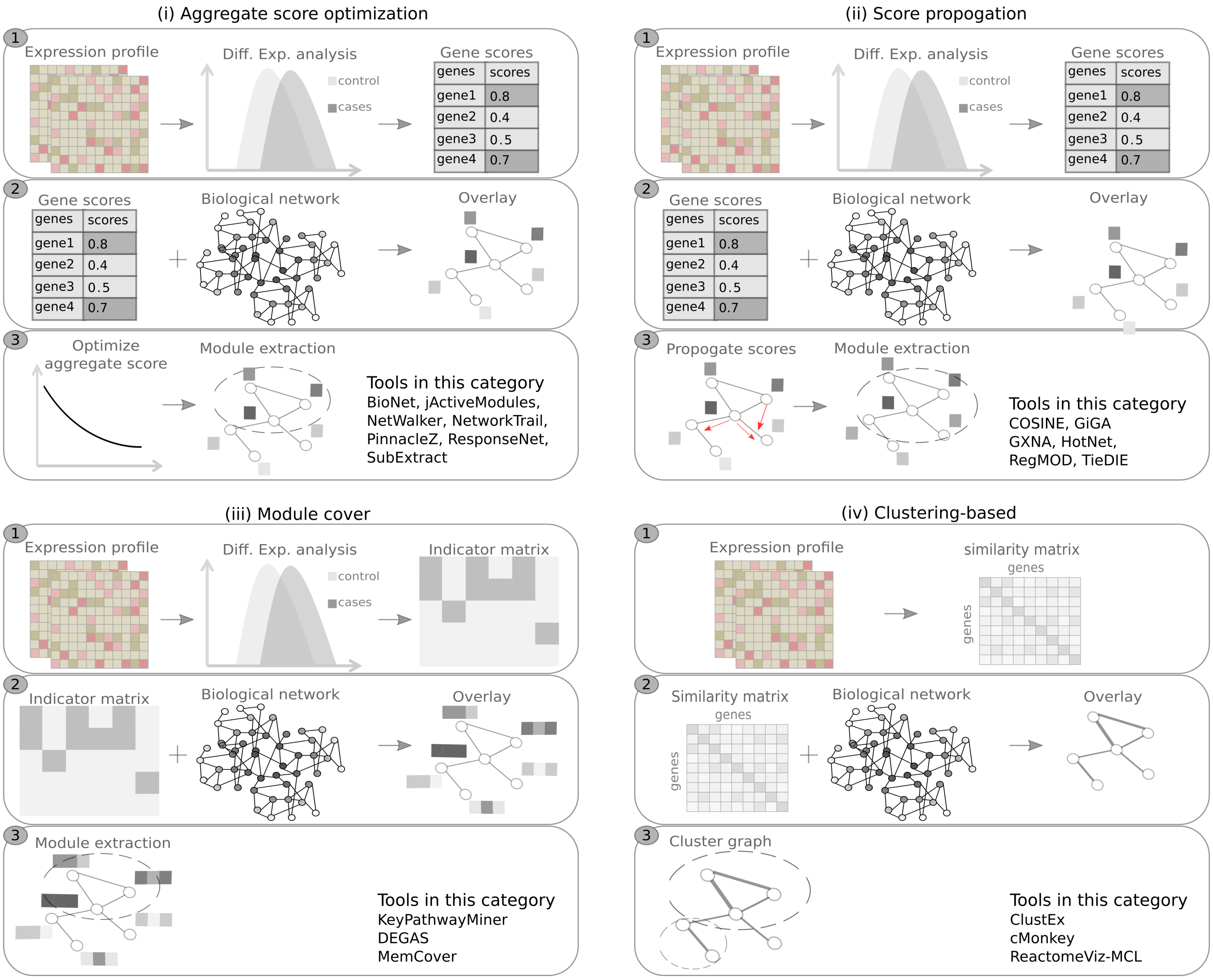

Supplementary Figure 1: Illustrations of the categories of algorithms

BioNet BioNet requires p-values as input, which can also be aggregated across different experiments. A beta uniform mixture distribution of the p-values is used to calculate maximum likelihood scores for each gene. BioNet's novelty lies in the next step, where an integer linear programming approach [29] is used to compute optimal sub-networks. This task is equivalent to solving the NP-hard maximal-scoring sub-graph problem and compute-intensive. Thus, BioNet additionally provides a heuristic that delivers an approximation of the optimal solution with less computational effort.

PinnacleZ In the aggregate score optimization method PinnacleZ [28] every gene in the network is used as a seed for a sub-network. These are iteratively extended as long as the new gene improves the total score. Each module is assigned a score, based on gene expression vectors in the module. In this way, PinnacleZ naturally creates a large number of sub-networks, which are subsequently filtered in three steps. Initially, the parameters of a null distribution are estimated based on the scores of all sub-networks. In this, either the normal distribution (t-test) or the gamma distribution (mutual information) is used. In the next step, PinnacleZ creates a number of random permutations of the gene labels. For each permutation, sub-networks are constructed and scored to construct a second null distribution. In the third step, another null distribution of scores is built based on sub-networks constructed after random permutations of phenotype labels. Sub-networks with a score that is significant in all three null distributions are reported as solutions.

COSINE In COSINE [45], both node and edge weights contribute to the final score in the extracted sub-network. Scores are calculated using the F-statistic for nodes and using the expected conditional F-statistic for edges, respectively. The latter serves as a measure of differential gene co-expression across different groups. COSINE relies on simulated annealing as a heuristic to find the sub-network that is optimal with respect to both of the scores.

GiGa In GiGA (Graph-based iterative Group Analysis), genes are first ranked based on their score in the experimental data. Subsequently, local minima are identified and used as starting points for iteratively building sub-networks with n members with a maximum rank m. Following a greedy approach, the neighboring gene with the lowest rank is added until all nodes with rank $\leq m$, that are reachable from the starting point are included. These sub-networks are then scored by calculating p-values for observing all n of n genes with rank m or lower in a list of all genes in the network.

GXNA GXNA (Gene eXpression Network Analysis) [46] is similar to GiGA but with an explicit focus on small sub-networks. Here, random nodes are selected as seeds of candidate sub-networks, which are iteratively extended by adding the neighboring node with the highest score (lowest rank in GiGA). The algorithm stops after a fixed sub-network size is reached or, alternatively, if the addition of an additional gene would decrease the total score of the sub-network. Random permutations of phenotype labels serve to assess the family-wise error rate. In contrast to GiGA, GXNA does not use the rank of a gene but an actual score, which can be computed by averaging the test

statistic or the gene expression values prior to performing the statistical test.

DEGAS / CUSP DEGAS is a pathway enrichment method integrated into the MATISSE tool suite [37]. MATISSE (Module Analysis via Topology of Interactions and Similarity SEts) is a tool suite with several algorithms for integrative analysis of networks and gene expression. The motivation of DEGAS is that in diseases, genes are not necessarily differentially expressed in all patients. Instead, one may observe that different genes are affected, which are, however, part of the same molecular pathway. In such cases, the goal of pathway enrichment should be to find sub-networks that are dysregulated in a disease by allowing different gene sets to be deregulated in each patient. This is the notion of the module cover approach. In addition to an interaction network, DEGAS requires an indicator matrix that specifies for each gene - case combination if that particular gene is differentially regulated ('1') or not ('0'). DEGAS then extracts all smallest possible sub-networks in which at least K genes are expressed in all but L cases. This is equivalent to the NP-hard set k-cover problem. The authors implemented a heuristic called CUSP (Covering Using Shortest Paths), which approximates the optimal solution.

KeyPathwayMiner KeyPathwayMiner is another module cover method that supports two similar yet distinct pathway enrichment strategies. In INES (Individual Node ExceptionS), two intuitive parameters are used to influence the size and composition of the extracted sub-networks. The first parameter defines whether a gene is considered as active, namely when it is active in all but L cases or samples. Whether a gene is active in a particular case or sample is defined by the user. The second parameter K corresponds to the number of exception genes that may be used to connect two otherwise disjoint but active sub-networks. While K allows users to conveniently extract larger solutions, it is prone to selecting hub genes. This behavior is not always desired, which is why the authors have devised a second strategy called GLONE (GLObal Node Exceptions) in which K is omitted in favor of a global view on L, which now defines that a sub-network is considered active if all of its genes are active in all but L cases or samples. KeyPathwayMiner provides an exact, a greedy as well as as ant colony heuristic to solve or approximate these NP-hard INES and GLONE problems.

4 Methods for generating evaluation datasets

Tool specific preprocessing of gene expression data

Gene expression data was sampled from a normal distribution with mean and variance as documented in Supplementary Table 3 for the varying mean scenario and in Supplementary Table 4 for the varying variance scenario, respectively. Data was generated for 110 samples constituting 100 cases, and 10 controls.

DEGAS, GXNA, PinnacleZ, use raw gene expression data and compute the significance internally. BioNet, takes the p-values and aggregates them internally. Thus, the case samples were Z transformed using mean and standard deviation of control samples. These z-scores were

converted to p-values by assuming normal distribution. KPM expects binary values ('1' if active and '0' otherwise). We thus generated a binary (indicator) matrix with a p-value cut-off of 0.05. Note that varying the p-value cut-off may yield different results. We did not this explore it in the current study. GiGA expects a ranked list of genes as input. Thus, the p-values were first ranked for each patient and subsequently the geometric mean was computed to determine the overall ranking of the genes. COSINE expects a single p-value per gene. Thus, we used the non-parametric wilcoxon-test to compare case and control samples.

AVD algorithms for placing FG nodes on the network

The AVD problem can be stated as under:

Problem 1 (AVD_K)**.** *Let $G = (V, E)$ be a connected graph, D be its (shortest) distance matrix, K, α be non-negative integers and N an integer with $N > 1$."*

Question: *Is there a subset $W \subseteq V$ with $|W| = N$ s.t.*

$$\mathrm{AVD}(W) := \frac{2}{N(N-1)} \sum_{i,j \in W} D_{ij} = K \pm \alpha$$

AVD_K problem is slightly different than *Maximum Diversity Problem* (*MDP*)[47] where $\alpha = 0$.In AVD_K, we need to account for the α parameter, which allows for a certain inexactness and variability in the solutions, i.e. the reported FG gene set. *Maximum Diversity Problem* (*MDP*), aims at finding an N element subset W of V such that the sum of pairwise distances of the elements contained in W is maximized. Kuo *et al.* showed that the (decision version of the) *MDP* is NP-complete which immediately implies that AVD_K is NP-complete as well. Hence, the computational complexity limits the exclusive use of exact techniques.

The AVD problem can be tackled with an integer liner programming (ILP) approach, but this is computationally expensive (in time and memory) for graphs with thousands of nodes and edges (see 6). To mitigate this issue, we developed a greedy heuristic to tackle the AVD_K problem (1). We start with an arbitrary vertex i and search for the next vertex j such that the distance $D_{i,j}$ is closest to K. This vertex j is added to $W(i)$ (Line 12). We then proceed to search for the next vertex j such that the average distance of the vertices in $W(i)$ and j gets again closest to K, and again j is added to $W(i)$. This step is repeated until $W(i)$ has N elements. We then remove $W(i)$ from V (Line 16) and repeat the procedure as long as all vertices are processed. The latter ensures that we get (greedily) a bunch of non-overlapping N-element subsets of V such that each such set W has average distance $\mathrm{AVD}(W) = K \pm \alpha$. As the runtime of this algorithms is dominated by the two for-loops (each runs at most $|V|$ times), the overall time complexity of the greedy approach is $O(|V|^2)$.

Algorithm 1 Greedy for AVD_K

INPUT: vertex set V, distance matrix D, integers $N,\ K,\ \alpha$;
Init $W(i) \leftarrow \{i\}$;
for each $i \in V$ **do**
  while $|W(i)| \neq N$ **do**
    $v_{\text{AVD}} \leftarrow 0$;
    for each $j \in V \setminus W(i)$ **do**
      if $\text{AVD}(W(i) \cup \{j\})$ is closer to K then v_{AVD} **then**
        $v_{\text{AVD}} \leftarrow \text{AVD}(W(i) \cup \{j\})$;
        $v \leftarrow j$;
      end if
    end for
    $W(i) \leftarrow W(i) \cup \{v\}$;
  end while
  if $\text{AVD}(W(i)) \in [K - \alpha, K + \alpha]$ **then**
    Save $W(i)$ as possible solution;
    $V \leftarrow V \setminus W(i)$; {to get non-overlapping sets}
  end if
end for
OUTPUT: Found solutions, i.e., non-overlapping sets W with $\text{AVD}(W) = K \pm \alpha$;

5 Supplementary Tables and Figures

Supplementary Table 1: *Range of internal parameter values used for each of the tools included in the comparative analysis*

Tool	Internal parameters	Range
BioNet	fdr (False Discovery Rate)	0.01, 0.05, 0.07, 0.1, 0.5
COSINE	minsize (Size of module)	10, 20, 30, 40, 50
DEGAS	k (noise)	1, 2, 3, 5, 10
GiGA	Size of module	10, 20, 30, 40, 50
GXNA	depth (Size of module)	10, 20, 30, 40, 50
KeyPathwayMiner (KPM)	L (exceptions)	250, 500, 1000, 1500, 2000
PinnacleZ	Size of module	10, 20, 30, 40, 50

Supplementary Table 2: *Non-default settings for additional tool parameters*

Tool	Non-default parameter values
BioNet	none
COSINE	none
DEGAS	-optalgo = 1 (for choosing CUSP algorithm from the menu)
GiGA	none
GXNA	-algoType=1 (for pathway enrichment)
KeyPathwayMiner (KPM)	Search algorithm: GREEDY, Strategy: GLONE
PinnacleZ	none

Supplementary Table 3: *Simulation Varying mean parameters: variance was set to 1 in all cases*

Signal strength	Mean of BG		Mean of FG	
	Case	Control	Case	Control
simulation control	0	0	0	0
low	0	0	2	1
medium	0	0	3	1
high	0	0	5	2

Supplementary Table 4: *Simulation Varying variance parameters; mean was set to zero in all cases*

Signal strength	Variance of BG		Variance of FG	
	Case	Control	Case	Control
simulation control	5	5	5	5
low	1	1	5	1
medium	0.5	0.5	5	0.5
high	0.2	0.2	5	0.2

Supplementary Table 5: *Numerical range of sparsity classes used to characterize the FG sets*

Sparsity	Algorithm	Low	Medium	High
Global FB Proximity				
	SAE	< 1.75	< 2.25	> 2.25
	AVD	< 2	< 3	> 3
Global FB Connectivity				
	SAE	< 2	< 5	> 5
	AVD	< 10	< 20	> 20
Local FB Density				
	SAE	< 0.2	< 0.3	> 0.3
	AVD	< 0.1	< 0.2	> 0.2

Supplementary Table 6: Running times of the two algorithms on an Intel® Xeon™ CPU with 3GHz. The parameters $\alpha = 2$ and the desired number of non-overlapping subset $m = 5$ were fixed for each test run. (OM = Out of memory)

$\|V\|$	$\|W\|$	K	Greedy	ILP
100	3	1	0m0.004s	0m7.757s
		2	0m0.004s	0m1.355s
1000	10	2	0m0.195s	OM
		3	0m0.188s	OM
5000	15	3	0m5.093s	OM
		4	0m5.097s	OM
		5	0m5.141s	OM
10000	20	4	0m23.155s	OM
		5	0m23.017s	OM
		6	0m23.791s	OM

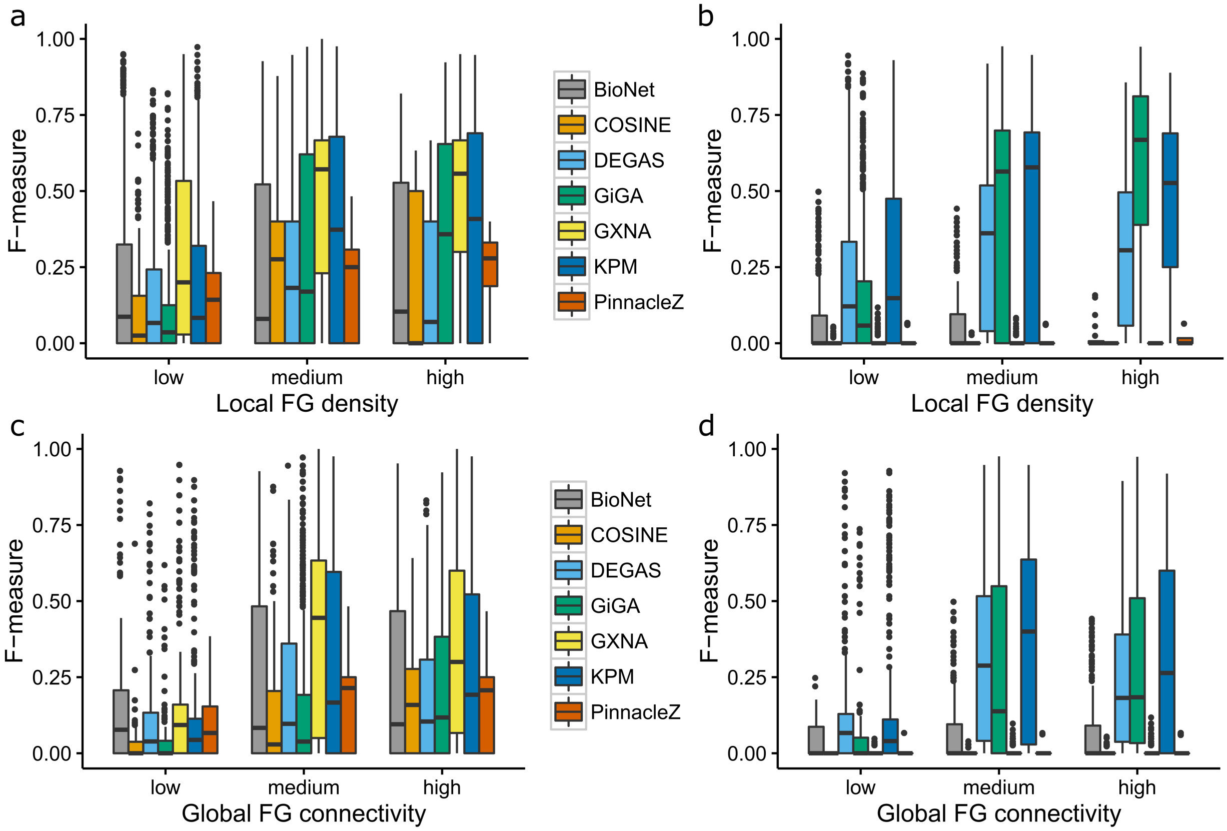

Supplementary Figure 2: Average performance for over 80 foreground (FG) sets of size n =20 generated using AVD_k algorithm with varying Local FG density (a, b) and varying Global FG connectivity (c, d). Expression profiles were simulated with varying mean (VM) (a, c) and varying variation (VV) (b, d). The HPRD network was used as input network. Performance is assessed using the F-measure in all cases. The error-bars (a, b) and box plots (c, d) represent performance over several FG nodes and over a range of internal parameter settings for each tool.

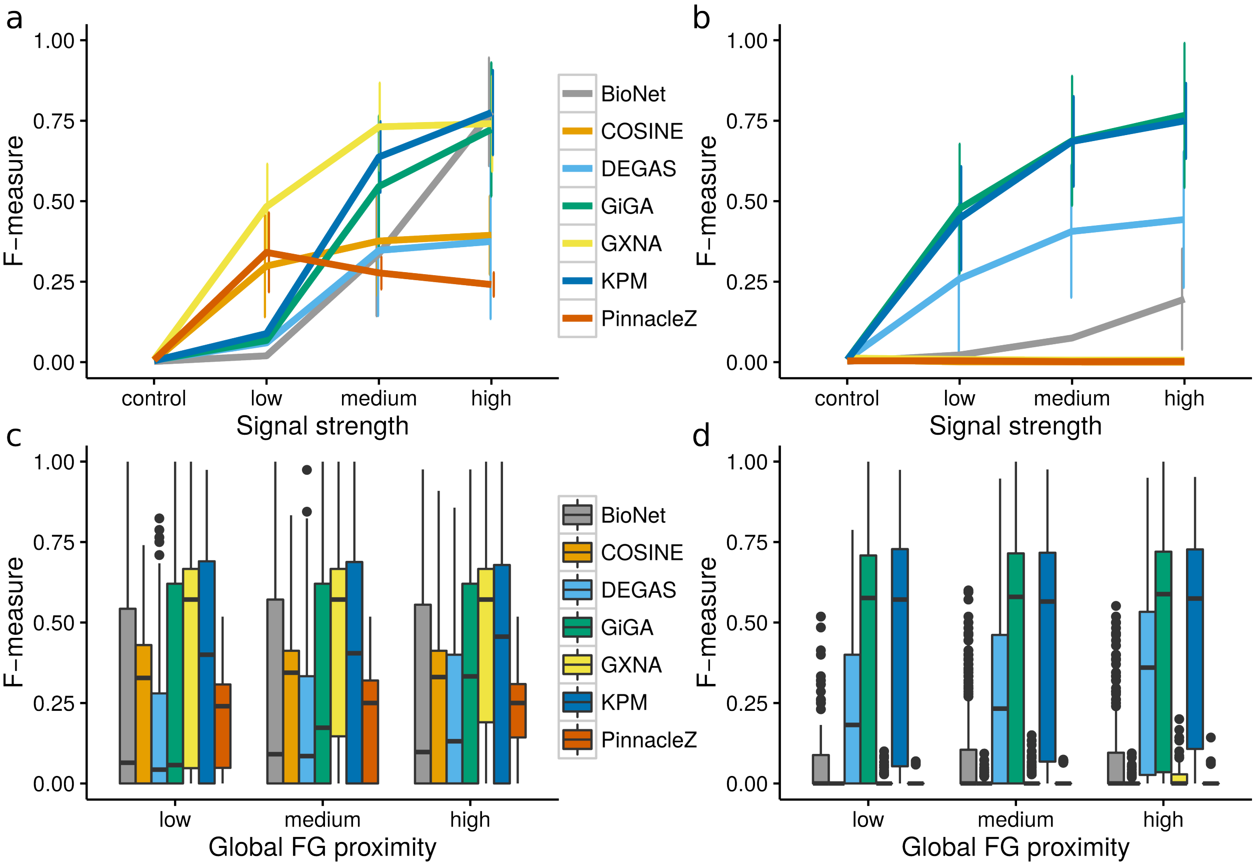

Supplementary Figure 3: Average performance for over 80 foreground (FG) sets of size n =20 generated using SAE algorithm with varying signal strength (a, b) and varying sparsity (c, d). Expression profiles were simulated with varying mean (VM) (a, c) and varying variation (VV) (b, d). The HPRD network was used as input network. Performance is assessed using the F-measure in all cases. The error-bars (a, b) and box plots (c, d) represent performance over several FG nodes and over a range of internal parameter settings for each tool.

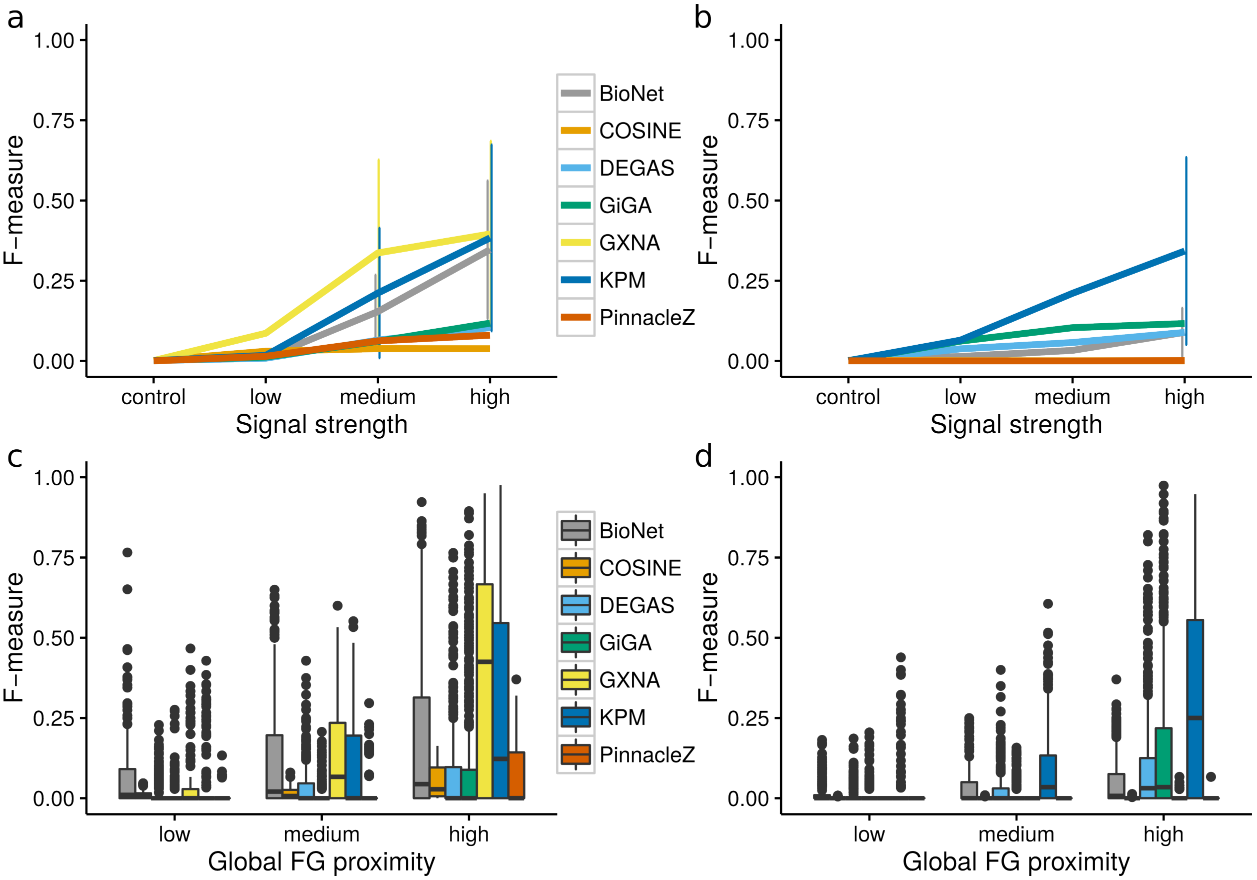

Supplementary Figure 4: Average performance for over 80 foreground (FG) sets of size n =20 generated using AVD_k algorithm with varying signal strength (a, b) and varying sparsity (c, d). Expression profiles were simulated with varying mean (VM) (a, c) and varying variation (VV) (b, d). The I2D network was used as input network. Performance is assessed using the F-measure in all cases. The error-bars (a, b) and box plots (c, d) represent performance over several FG nodes and over a range of internal parameter settings for each tool.

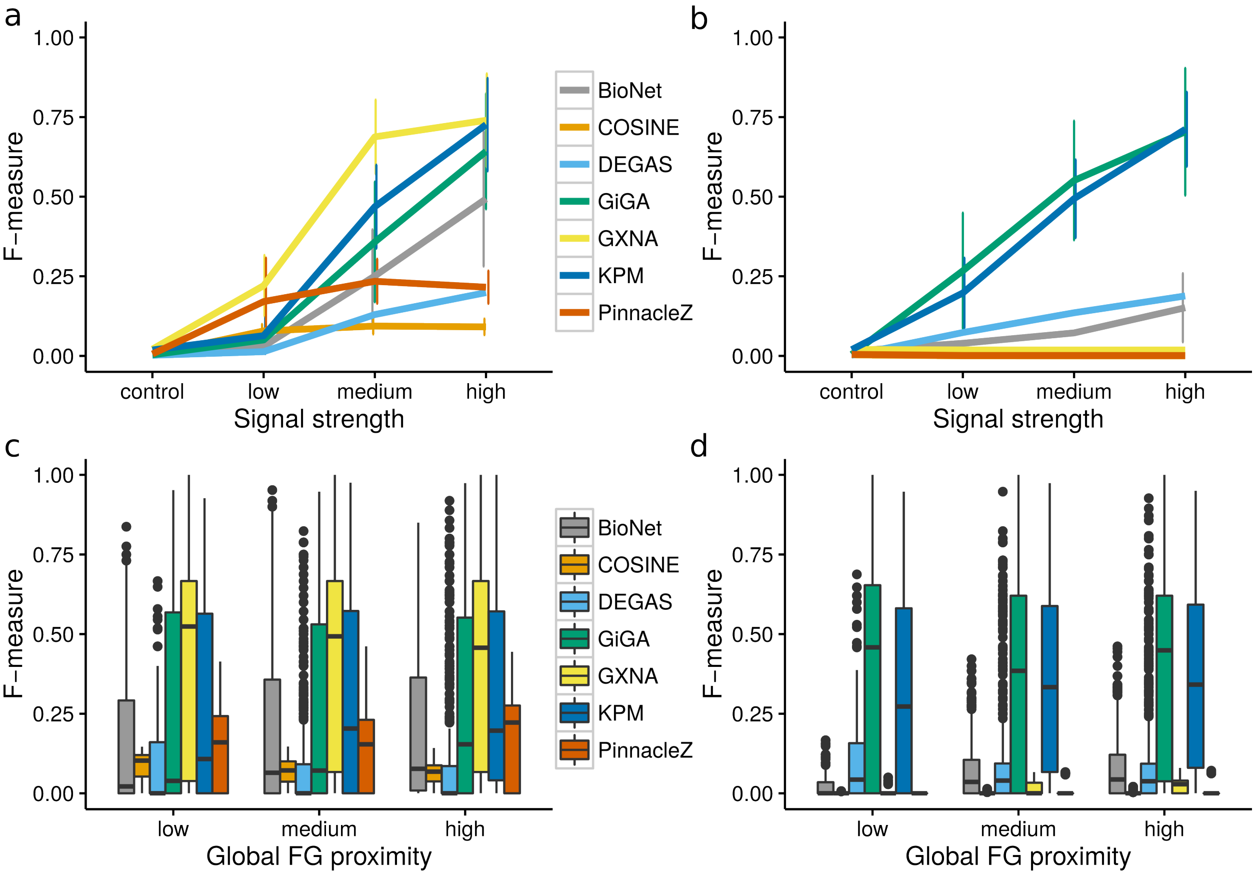

Supplementary Figure 5: Average performance for over 80 foreground (FG) sets of size n =20 generated using SAE algorithm with varying signal strength (a, b) and varying sparsity (c, d). Expression profiles were simulated with varying mean (VM) (a, c) and varying variation (VV) (b, d). The I2D network was used as input network. Performance is assessed using the F-measure in all cases. The error-bars (a, b) and box plots (c, d) represent performance over several FG nodes and over a range of internal parameter settings for each tool.

References

1. Ashburner, M. *et al.* Gene ontology: tool for the unification of biology. The Gene Ontology Consortium. *Nature genetics* **25**, 25–29 (2000). 10614036.

2. Subramanian, A. *et al.* Gene set enrichment analysis: a knowledge-based approach for interpreting genome-wide expression profiles. *Proc Natl Acad Sci U S A* **102**, 15545–15550 (2005).

3. Kanehisa, M. & Goto, S. Kegg: kyoto encyclopedia of genes and genomes. *Nucleic Acids Res* **28**, 27–30 (2000).

4. Croft, D. *et al.* Reactome: a database of reactions, pathways and biological processes. *Nucleic Acids Res* **39**, D691–D697 (2011).

5. Caspi, R. *et al.* The metacyc database of metabolic pathways and enzymes and the biocyc collection of pathway/genome databases. *Nucleic Acids Res* **44**, D471–D480 (2016).

6. Falcon, S. & Gentleman, R. Using gostats to test gene lists for go term association. *Bioinformatics* **23**, 257–258 (2007).

7. Huang, D. W. *et al.* David bioinformatics resources: expanded annotation database and novel algorithms to better extract biology from large gene lists. *Nucleic Acids Res* **35**, W169–W175 (2007).

8. Mi, H., Muruganujan, A., Casagrande, J. T. & Thomas, P. D. Large-scale gene function analysis with the panther classification system. *Nat Protoc* **8**, 1551–1566 (2013).

9. Kamburov, A., Stelzl, U., Lehrach, H. & Herwig, R. The consensuspathdb interaction database: 2013 update. *Nucleic Acids Res* **41**, D793–D800 (2013).

10. Barry, W. T., Nobel, A. B. & Wright, F. A. Significance analysis of functional categories in gene expression studies: a structured permutation approach. *Bioinformatics* **21**, 1943–1949 (2005).

11. Luo, W., Friedman, M. S., Shedden, K., Hankenson, K. D. & Woolf, P. J. Gage: generally applicable gene set enrichment for pathway analysis. *BMC Bioinformatics* **10**, 161 (2009).

12. Michaud, J. *et al.* Integrative analysis of runx1 downstream pathways and target genes. *BMC Genomics* **9**, 363 (2008).

13. Barbie, D. A. *et al.* Systematic rna interference reveals that oncogenic kras-driven cancers require tbk1. *Nature* **462**, 108–112 (2009).

14. Hänzelmann, S., Castelo, R. & Guinney, J. Gsva: gene set variation analysis for microarray and rna-seq data. *BMC Bioinformatics* **14**, 7 (2013).

15. Tarca, A. L., Bhatti, G. & Romero, R. A comparison of gene set analysis methods in terms of sensitivity, prioritization and specificity. *PLoS One* **8**, e79217 (2013).

16. Gao, S. & Wang, X. Tappa: topological analysis of pathway phenotype association. *Bioinformatics* **23**, 3100–3102 (2007).

17. Hung, J.-H. *et al.* Identification of functional modules that correlate with phenotypic difference: the influence of network topology. *Genome Biol* **11**, R23 (2010).

18. Köhler, S., Bauer, S., Horn, D. & Robinson, P. N. Walking the interactome for prioritization of candidate disease genes. *Am J Hum Genet* **82**, 949–958 (2008).

19. Li, Y. & Patra, J. C. Integration of multiple data sources to prioritize candidate genes using discounted rating system. *BMC Bioinformatics* **11 Suppl 1**, S20 (2010).

20. Liu, W. *et al.* Topologically inferring risk-active pathways toward precise cancer classification by directed random walk. *Bioinformatics* **29**, 2169–2177 (2013).

21. Tarca, A. L. *et al.* A novel signaling pathway impact analysis. *Bioinformatics* **25**, 75–82 (2009).

22. Page, L., Brin, S., Motwani, R. & Winograd, T. The pagerank citation ranking: bringing order to the web. (1999).

23. Vaske, C. J. *et al.* Inference of patient-specific pathway activities from multi-dimensional cancer genomics data using PARADIGM. *Bioinformatics (Oxford, England)* **26**, i237–45 (2010).

24. De Smet, R. & Marchal, K. Advantages and limitations of current network inference methods. *Nat Rev Microbiol* **8**, 717–729 (2010).

25. Ideker, T., Ozier, O., Schwikowski, B. & Siegel, A. F. Discovering regulatory and signalling circuits in molecular interaction networks. *Bioinformatics (Oxford, England)* **18 Suppl 1**, S233–S240 (2002).

26. Guo, Z. *et al.* Edge-based scoring and searching method for identifying condition-responsive protein-protein interaction sub-network. *Bioinformatics* **23**, 2121–2128 (2007).

27. Cabusora, L., Sutton, E., Fulmer, A. & Forst, C. V. Differential network expression during drug and stress response. *Bioinformatics* **21**, 2898–2905 (2005).

28. Chuang, H.-Y., Lee, E., Liu, Y.-T., Lee, D. & Ideker, T. Network-based classification of breast cancer metastasis. *Mol Syst Biol* **3**, 140 (2007).

29. Dittrich, M. T., Klau, G. W., Rosenwald, A., Dandekar, T. & Müller, T. Identifying functional modules in protein–protein interaction networks: an integrated exact approach. *Bioinformatics* **24**, i223–i231 (2008).

30. Hwang, T. & Park, T. Identification of differentially expressed subnetworks based on multivariate anova. *BMC Bioinformatics* **10**, 128 (2009).

31. Klammer, M., Godl, K., Tebbe, A. & Schaab, C. Identifying differentially regulated subnetworks from phosphoproteomic data. *BMC Bioinformatics* **11**, 351 (2010).

32. Backes, C. *et al.* An integer linear programming approach for finding deregulated subgraphs in regulatory networks. *Nucleic Acids Res* **40**, e43 (2012).

33. Vandin, F., Upfal, E. & Raphael, B. J. Algorithms for detecting significantly mutated pathways in cancer. *J Comput Biol* **18**, 507–522 (2011).

34. Komurov, K., Dursun, S., Erdin, S. & Ram, P. T. Netwalker: a contextual network analysis tool for functional genomics. *BMC Genomics* **13**, 282 (2012).

35. Richardson, M. & Domingos, P. The intelligent surfer: Probabilistic combination of link and content information in pagerank (2002).

36. Qiu, Y.-Q., Zhang, S., Zhang, X.-S. & Chen, L. Detecting disease associated modules and prioritizing active genes based on high throughput data. *BMC Bioinformatics* **11**, 26 (2010).

37. Ulitsky, I., Karp, R. & Shamir, R. Detecting disease-specific disregulated pathways via analysis of clinical expression profiles. *Proceedings of RECOMB, Research in Computational Molecular Biology* **4955**, 347–359 (2008).

38. Alcaraz, N. *et al.* Efficient key pathway mining: combining networks and OMICS data. *Integrative biology : quantitative biosciences from nano to macro* **4**, 756–64 (2012).

39. Kim, Y.-A., Salari, R., Wuchty, S. & Przytycka, T. M. Module cover - a new approach to genotype-phenotype studies. *Pac Symp Biocomput* 135–146 (2013).

40. Gu, J., Chen, Y., Li, S. & Li, Y. Identification of responsive gene modules by network-based gene clustering and extending: application to inflammation and angiogenesis. *BMC Syst Biol* **4**, 47 (2010).

41. Wu, G. & Stein, L. A network module-based method for identifying cancer prognostic signatures. *Genome Biol* **13**, R112 (2012).

42. Prelić, A. *et al.* A systematic comparison and evaluation of biclustering methods for gene expression data. *Bioinformatics* **22**, 1122–1129 (2006).

43. Chen, R. *et al.* Personal omics profiling reveals dynamic molecular and medical phenotypes. *Cell* **148**, 1293–1307 (2012).

44. Hanisch, D., Zien, A., Zimmer, R. & Lengauer, T. Co-clustering of biological networks and gene expression data. *Bioinformatics* **18 Suppl 1**, S145–S154 (2002).

45. Ma, H., Schadt, E. E., Kaplan, L. M. & Zhao, H. Cosine: Condition-specific sub-network identification using a global optimization method. *Bioinformatics* **27**, 1290–1298 (2011).

46. Nacu, S., Critchley-Thorne, R., Lee, P. & Holmes, S. Gene expression network analysis and applications to immunology. *Bioinformatics* **23**, 850–858 (2007).

47. Kuo, C.-C., Glover, F. & Dhir, K. S. Analyzing and modeling the maximum diversity problem by zero-one programming. *Decision Sciences* **24**, 1171–1185 (1993).
